# Supplementary material for: Post-Fracture Inpatient and Outpatient Physical/Occupational Therapy and Its Association with Survival among Adults with Cerebral Palsy
Source: J Clin Med. 2022 Sep 22;11(19):5561. doi: 10.3390/jcm11195561 (PMC9570908; doi:10.3390/jcm11195561)

Supplementary Table S1. International Classification of Diseases, Ninth (ICD-9) and Tenth (ICD-10) Revision, Clinical Modification, Healthcare Common Procedure Coding System (HCPCS), Current Procedural Terminology (CPT), and Revenue codes to identify variables for this study.

	ICD-9 codes	ICD-10 codes
**Cerebral palsy**	333.71, 343.0–343.4, 343.8, 343.9	G80.x
**Whitney Comorbidity Index comorbidities**		
Hypertension (un)complicated	401., 401.9, 402.10, 402.90, 404.10, 404.90, 405.11, 405.19, 405.91, 405.99	I10.x, I11.x-I13.x, I15.x
Epilepsy	345.x	G40.x
Chronic pulmonary disease	416.8, 416.9, 490.x-505.x, 506.4, 508.1, 508.8, 518.83	I27.8, I27.9, J40.x-J47.x, J60.x-J67.x, J68.4, J70.1, J70.3, J96.10
Depression	300.4, 301.12, 309.0, 309.1, 311	F20.4, F31.3-F31.5, F32.x, F33.x, F34.1, F41.2, F43.2
Blood loss and deficiency anemias	280.x-281.9, 285.9	D50.0, D50.8, D50.9, D51.x-D53.x
Gastrointestinal issues	531.70, 531.90, 532.70, 532.90, 533.70, 533.90, 534.70, 534.90, 556.x, 564.0x, 564.1, V12.71	K25.7, K25.9, K26.7, K26.9, K27.7, K27.9, K28.7, K28.9, K50.x-K52.x, K59.0, K58.x
Osteoarthritis and allied disorders	715.x	M15.x-M19.x
Intellectual disabilities	317.x-319.x	F70.x-F73.x, F78.x, F79.x
Fluid and electrolyte disorders	276.0–276.9	E22.2, E86.x, E87.x
Cardiac arrhythmias	426.10, 426.11, 426.13, 426.2–426.53, 426.6–426.89, 427.0, 427.2, 427.31, 427.60, 427.9, 785.0, V45.0, V53.3	I44.1-I44.3, I45.6, I45.9, I47.x-I49.x, R00.0, R00.1, R00.8, T82.1, Z45.0, Z95.0
Hypothyroidism	243–244.2, 244.8, 244.9	E00.x-E03.x, E89.0
Dysphagia	787.2x	R13.1x
Cerebrovascular disease	362.34, 430.x-438.x	G45.x, G46.x, H34.0, I60.x-I69.x
Pneumonia	480.x-486.x	J13, J14, J15.x, J16.x, J17, J18.x
Diabetes without chronic complication	250.0–250.3, 250.8, 250.9	E10.0, E10.1, E10.6, E10.8, E10.9, E11.0, E11.1, E11.6, E11.8, E11.9, E12.0, E12.1, E12.6, E12.8, E12.9, E13.0, E13.1, E13.6, E13.8, E13.9, E14.0, E14.1, E14.6, E14.8, E14.9
Other neurological disorders	331.9, 332.0, 333.4, 333.5, 334.0–335.9, 340, 341.1–341.9, 348.1, 348.3, 780.3, 784.3	G10.x-G13.x, G20.x-G22.x, G25.4, G25.5, G31.2, G31.8, G31.9, G32.x, G35.x-G37.x, G41.x, G93.1, G93.4, R47.0, R56.x
Renal disease	403.01, 403.11, 403.91, 404.02, 404.03, 404.12, 404.13, 404.92,	I12.0, I13.1, N03.2-N03.7, N05.2-N05.7, N18.x, N19.x,

	404.93, 582.x, 583.0–583.7, 585.x, 586.x, 588.0, V42.0, V45.1, V56.x	N25.0, Z49.0-Z49.2, Z94.0, Z99.2
Any malignancy, including lymphoma and leukemia, except malignant neoplasm of skin	140.x-172.x, 174.x-195.8, 200.x-208.x, 238.6, V10.00-V10.9	C00.x-C26.x, C30.x-C34.x, C37.x-C41.x, C43.x, C45.x-C58.x, C60.x-C76.x, C81.x-C85.x, C88.x, C90.x-C97.x
Congestive heart failure	398.91, 402.11, 402.91, 404.11, 404.13, 404.91, 404.93, 428.0–428.9	I09.9, I11.0, I13.0, I13.2, I25.5, I42.0, I42.5-I42.9, I43.x, I50.x, P29.0
Diabetes with chronic complication	250.4–250.7	E10.2-E10.5, E10.7, E11.2-E11.5, E11.7, E12.2-E12.5, E12.7, E13.2-E13.5, E13.7, E14.2-E14.5, E14.7
Neurogenic bowel or bladder	564.81, 596.54	K59.2, N31.x
Mild to severe liver disease	070.22, 070.23, 070.32, 070.33, 070.44, 070.54, 070.6, 070.9, 456.0–456.2, 570.x, 571.x, 572.2–572.8, 573.3, 573.4, 573.8, 573.9, V42.7	B18.x, K70.0-K70.3, K70.9, K71.3-K71.5, K71.7, K73.x, K74.x, K76.0, K76.2-K76.4, K76.8, K76.9, Z94.4, I85.0, I85.9, I86.4, I98.2, K70.4, K71.1, K72.1, K72.9, K76.5, K76.6, K76.7
Dementia	290.x, 294.1, 331.0, 331.2	F00.x-F03.x, F05.1, G30.x, G31.1
Myocardial infarction	410.x, 412.x	I21.x, I22.x, I25.2
Rheumatoid arthritis and other inflammatory polyarthropathies	714.x	M05.x, M06.x, M08.0-M08.48
Metastatic cancer	196.x-199.1	C77.x-C80.x

	**HCPCS codes**	**CPT codes**	**Revenue**
**Outpatient physical and occupational therapy**			
Physical therapy-related services	G0151, G0157, G0159, G0237-G0239, G0422-G0424, G2168	97010, 97014, 97022, 97024, 97026, 97035, 97039, 97110, 97140, 97150, 97535	0420–0423, 0429
Occupational therapy-related services	G0129, G0152, G0158, G0160, G2169	97537, 97545, 97546	0430–0433, 0439
Physical or occupational therapy, not distinguishable	G0176	97012, 97016, 97018, 97028, 97032, 97033, 97034, 97036, 97112, 97113, 97116, 97124, 97139, 97530, 97542, 97799	

Supplementary Table S2. Adjusted marginal means and cost ratio for U.S. region and division of residence with the outcome as average cost per day for physical/occupational therapy* during the post-fracture acute care/rehabilitation stay (*n* = 649).

	Marginal means ($) (95% CI)	Cost ratio (95% CI)
**U.S. region of residence**		
Northeast	2,034 (1,464, 2,826)	
Midwest	2,008 (1,453, 2,773)	
South	2,001 (1,474, 2,717)	
West	2,115 (1,564, 2,858)	
Northeast vs. Midwest		1.01 (0.83, 1.24)
Northeast vs. South		1.02 (0.84, 1.23)
Northeast vs. West		0.96 (0.78, 1.19)
Midwest vs. South		1.00 (0.83, 1.21)
Midwest vs. West		0.95 (0.77, 1.17)
South vs. West		0.95 (0.78, 1.15)
**U.S. division of residence**		
New England	1,925 (1,250, 2,963)	
Middle Atlantic	2,090 (1,491, 2,930)	
East North-central	2,053 (1,469, 2,871)	
West North-central	1,925 (1,293, 2,865)	
South Atlantic	1,939 (1,396, 2,694)	
East South-central	1,802 (1,235, 2,630)	
West South-central	2,310 (1,609, 3,317)	
Mountain	2,182 (1,477, 3,223)	
Pacific	2,099 (1,538, 2,865)	
New England vs. Middle Atlantic		0.92 (0.65, 1.31)
New England vs. East North-central		0.94 (0.66, 1.33)
New England vs. West North-central		1.00 (0.67, 1.50)
New England vs. South Atlantic		0.99 (0.70, 1.40)
New England vs. East South-central		1.07 (0.71, 1.60)
New England vs. West South-central		0.83 (0.57, 1.21)
New England vs. Mountain		0.88 (0.59, 1.33)
New England vs. Pacific		0.92 (0.64, 1.32)
Middle Atlantic vs. East North-central		1.02 (0.80, 1.29)
Middle Atlantic vs. West North-central		1.09 (0.79, 1.49)
Middle Atlantic vs. South Atlantic		1.08 (0.84, 1.38)
Middle Atlantic vs. East South-central		1.16 (0.85, 1.57)
Middle Atlantic vs. West South-central		0.90 (0.68, 1.20)
Middle Atlantic vs. Mountain		0.96 (0.70, 1.31)

Middle Atlantic vs. Pacific	1.00 (0.77, 1.28)
East North-central vs. West North-central	1.07 (0.78, 1.46)
East North-central vs. South Atlantic	1.06 (0.83, 1.34)
East North-central vs. East South-central	1.14 (0.84, 1.54)
East North-central vs. West South-central	0.89 (0.67, 1.17)
East North-central vs. Mountain	0.94 (0.69, 1.29)
East North-central vs. Pacific	0.98 (0.76, 1.26)
West North-central vs. South Atlantic	0.99 (0.72, 1.36)
West North-central vs. East South-central	1.07 (0.74, 1.54)
West North-central vs. West South-central	0.83 (0.59, 1.18)
West North-central vs. Mountain	0.88 (0.61, 1.29)
West North-central vs. Pacific	0.92 (0.66, 1.27)
South Atlantic vs. East South-central	1.08 (0.79, 1.47)
South Atlantic vs. West South-central	0.84 (0.64, 1.10)
South Atlantic vs. Mountain	0.89 (0.65, 1.22)
South Atlantic vs. Pacific	0.92 (0.71, 1.19)
East South-central vs. West South-central	0.78 (0.56, 1.09)
East South-central vs. Mountain	0.83 (0.57, 1.20)
East South-central vs. Pacific	0.86 (0.63, 1.18)
West South-central vs. Mountain	1.06 (0.75, 1.50)
West South-central vs. Pacific	1.10 (0.82, 1.47)
Mountain vs. Pacific	1.04 (0.75, 1.44)

CI, confidence interval. *Findings were similar when physical and occupational therapy were examined separately. The models are adjusted for age, gender, race, dual eligibility with Medicaid, the Whitney Comorbidity Index, acute care/rehabilitation length of stay, and fracture site.

Supplementary Table S3. Prevalence of Whitney Comorbidity Index comorbidities for adults with cerebral palsy with a fragility fracture that were admitted to an acute care/rehabilitation setting (*n* = 649).

	**% (*n*)**
Hypertension	42.8 (278)
Other neurological disorders, excluding epilepsy	31.7 (206)
Epilepsy	30.0 (195)
Intellectual disabilities	28.5 (185)
Gastrointestinal issues	27.1 (176)
Fluid and electrolyte disorders	26.2 (170)
Osteoarthritis	24.2 (157)
Depression	22.2 (144)
Cardiac arrhythmias	22.2 (144)
Blood loss anemia	21.7 (141)
Chronic pulmonary disease	20.6 (134)
Hypothyroidism	19.9 (129)
Diabetes without complications	18.6 (121)
Dysphagia	18.0 (117)
Pneumonia	15.6 (101)
Cerebrovascular disease	15.4 (100)
Congestive heart failure	10.9 (71)
Renal disease	9.9 (64)
Any malignancy, including lymphoma and leukemia, except malignant neoplasm of skin	9.9 (64)
Neurogenic bowel or bladder	9.6 (62)
Diabetes with complications	9.1 (59)
Dementia/Alzheimer disease	6.3 (41)
Liver disease	5.5 (36)
Myocardial infarction	2.9 (19)
Rheumatoid arthritis	2.5 (16)
Metastatic cancer	1.5 (10)

Supplementary Figure S1. Flow chart to derive the analytic sample of adults with cerebral palsy (CP).

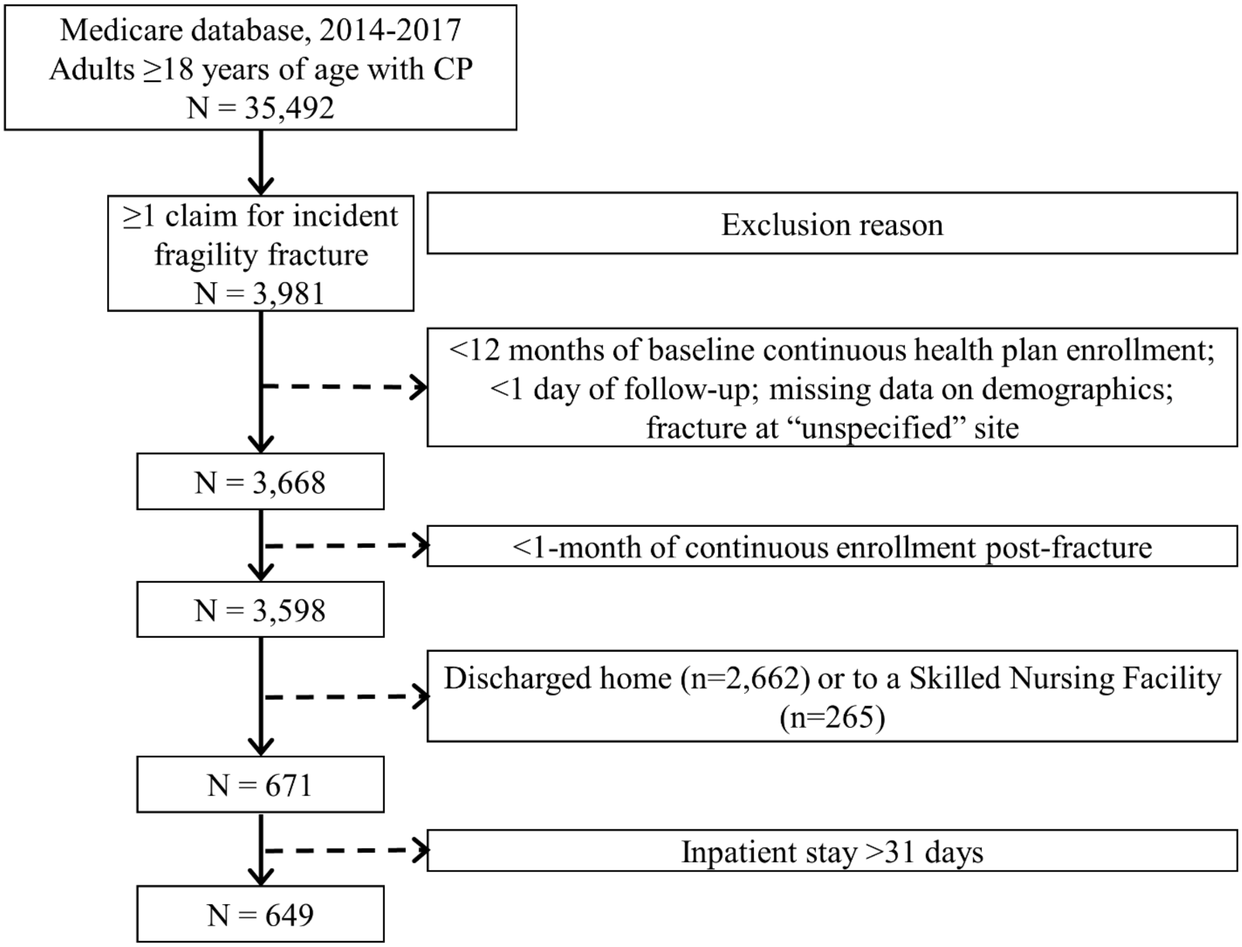

Supplementary Figure S2. Therapy volume by length of stay effect on mortality. Examining the interaction between the acute care/rehabilitation average physical/occupational therapy (PT/OT) cost/day and length of stay for the outcome, 1-year mortality rate, among adults with cerebral palsy with an incident fragility fracture, after additionally adjusting for outpatient PT/OT. The open and closed circles represent the hazard ratio (vertical lines are the 95% confidence interval) of mortality (y-axis) for the average physical/occupational therapy cost/day for that length of stay at 1, 3, 5, and every other day until 29 days (x-axis). The same length of stay was estimated for the unadjusted and adjusted models, but positioned next to one another on the graph as opposed to on top of one another to enhance visual interpretation. If the 95% confidence interval (vertical lines extending from the open/closed circles) cross 1.00 (dashed line), the association is not statistically significant at $p < 0.05$. The adjusted model accounted for age, the Whitney Comorbidity Index, and outpatient PT/OT.

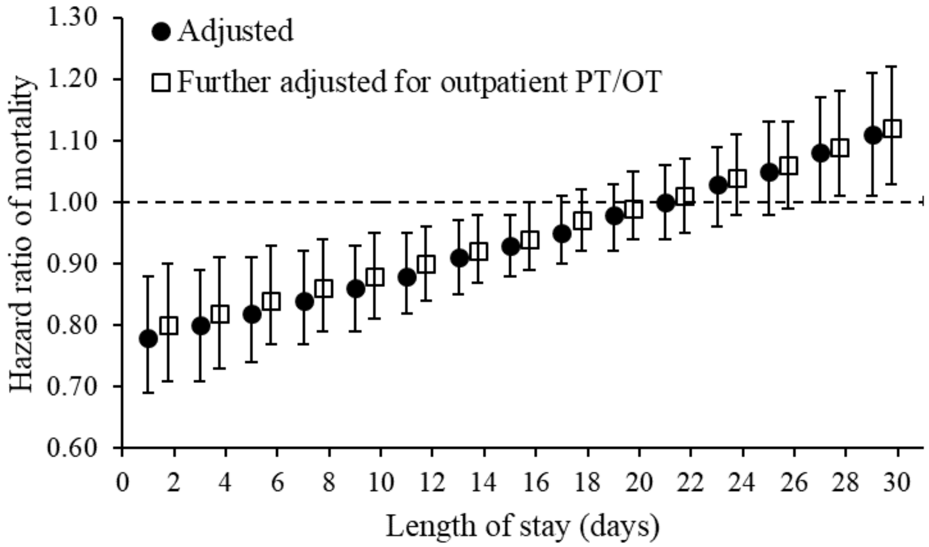

Supplement: Supplementary file 1 [file jcm-11-05561-s001.zip › jcm-1897790-supplementary.pdf]
